# Supplementary material for: Therapeutic Effects of Perilla Phenols in Oral Squamous Cell Carcinoma
Source: Int J Mol Sci. 2023 Oct 5;24(19):14931. doi: 10.3390/ijms241914931 (PMC10573788; doi:10.3390/ijms241914931)
Supplement: Supplementary file 1 [file ijms-24-14931-s001.zip › ijms-2617103-supplementary.pdf]

Supplementary Data

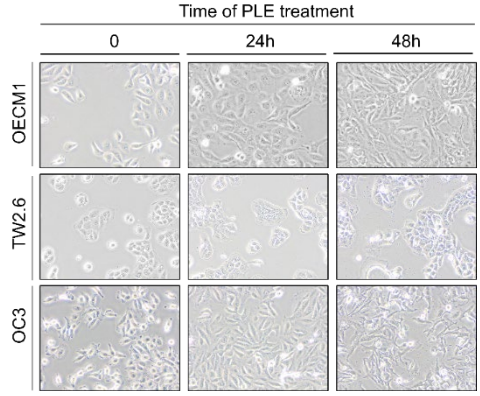

Figure S1. Sensitivity of OSCC to PLE assessed by cell morphology. The representative images of OSCC cell lines before and after 4 mg/mL PLE treatment were taken at indicated times by Olympus CKX53 inverted microscope.

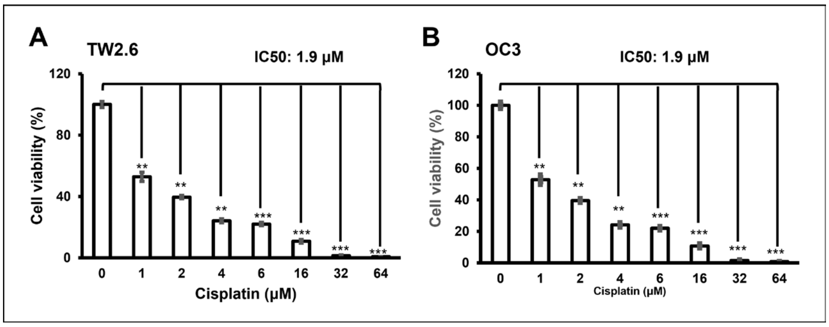

Figure S2. IC_{50} of cisplatin for TW2.6 and OC3 cells. Cell viability was determined by MTT assay. Data are presented as mean ± SEM of at least three independent experiments (**$p < 0.01$, ***$p < 0.005$)

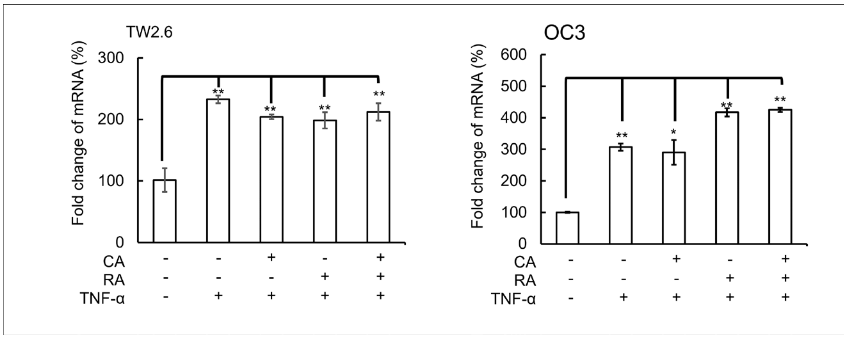

Figure S3. Effect of CA and RA on the expression of IL-1b mRNA. The levels of IL-1b expression were determined by quantitative PCR with TW2.6 and OC3 cells under the conditions as described in Figure 4b. Data are presented as mean ± SEM of three independent experiments (*$p < 0.05$, **$p < 0.01$).
